# Automated SPECT analysis compared with expert visual scoring for the detection of FFR-defined coronary artery disease

**DOI:** 10.1007/s00259-018-3951-1

**Published:** 2018-02-22

**Authors:** R. S. Driessen, P. G. Raijmakers, I. Danad, W. J. Stuijfzand, S. P. Schumacher, J.A. Leipsic, J. K. Min, J. Knuuti, A. A. Lammertsma, A. C. van Rossum, N. van Royen, S. R. Underwood, P. Knaapen

**Affiliations:** 1 Department of Cardiology, VU University Medical Center, De Boelelaan 1117, 1081 HV Amsterdam, The Netherlands; 2 Department of Radiology & Nuclear Medicine, VU University Medical Center, Amsterdam, The Netherlands; 3 Department of Radiology, St. Paul’s Hospital, Vancouver, Canada; 4 Department of Radiology, Weill Cornell Medical College, New York-Presbyterian Hospital, New York, USA; 5 Turku University Hospital and University of Turku, Turku, Finland; 6 Department of Nuclear Medicine, Royal Brompton Hospital, London, UK

**Keywords:** Myocardial perfusion imaging, SPECT, Automated analysis, Coronary artery disease, Ischemia

## Abstract

**Purpose:**

Traditionally, interpretation of myocardial perfusion imaging (MPI) is based on visual assessment. Computer-based automated analysis might be a simple alternative obviating the need for extensive reading experience. Therefore, the aim of the present study was to compare the diagnostic performance of automated analysis with that of expert visual reading for the detection of obstructive coronary artery disease (CAD).

**Methods:**

206 Patients (64% men, age 58.2 ± 8.7 years) with suspected CAD were included prospectively. All patients underwent ^99m^Tc-tetrofosmin single-photon emission computed tomography (SPECT) and invasive coronary angiography with fractional flow reserve (FFR) measurements. Non-corrected (NC) and attenuation-corrected (AC) SPECT images were analyzed both visually as well as automatically by commercially available SPECT software. Automated analysis comprised a segmental summed stress score (SSS), summed difference score (SDS), stress total perfusion deficit (S-TPD), and ischemic total perfusion deficit (I-TPD), representing the extent and severity of hypoperfused myocardium. Subsequently, software was optimized with an institutional normal database and thresholds. Diagnostic performances of automated and visual analysis were compared taking FFR as a reference.

**Results:**

Sensitivity did not differ significantly between visual reading and most automated scoring parameters, except for SDS, which was significantly higher than visual assessment (*p* < 0.001). Specificity, however, was significantly higher for visual reading than for any of the automated scores (*p* < 0.001 for all). Diagnostic accuracy was significantly higher for visual scoring (77.2%) than for all NC images scores (*p* < 0.05), but not compared with SSS AC and S-TPD AC (69.8% and 71.2%, *p* = 0.063 and *p* = 0.134). After optimization of the automated software, diagnostic accuracies were similar for visual (73.8%) and automated analysis. Among the automated parameters, S-TPD AC showed the highest accuracy (73.5%).

**Conclusion:**

Automated analysis of myocardial perfusion SPECT can be as accurate as visual interpretation by an expert reader in detecting significant CAD defined by FFR.

**Electronic supplementary material:**

The online version of this article (10.1007/s00259-018-3951-1) contains supplementary material, which is available to authorized users.

## Introduction

Myocardial perfusion imaging (MPI) using single-photon emission computed tomography (SPECT) is widely used for non-invasive diagnosis of obstructive coronary artery disease (CAD) [1]. In general, SPECT studies are graded based on visual assessment of relative tracer uptake images with subjective interpretation that includes factors such as pre-test likelihood of disease, image quality, and potential (attenuation) artifacts. Expert reading with comprehensive evaluation of these factors requires prolonged periods of training, but ultimately it remains subjective. To assist clinical decision-making, commercially available software packages have been developed to analyze MPI images based on normal databases [2–4]. Data on the actual clinical diagnostic performance of automated MPI SPECT analysis are, however, scarce [5–7]. Therefore, current guidelines recommend that automated analysis should be used only as an adjunct to visual analysis [8]. It is important to note that these recommendations have been based primarily on relatively outdated SPECT MPI technology without currently available attenuation correction (AC) or appropriate validation against valid reference standards [5–7, 9]. As a reference standard, predominantly invasive coronary angiography (ICA) is used, despite the fact that only fractional flow reserve (FFR) guided treatment has shown improvement in event-free survival and its frequent discrepancy with angiography is increasingly acknowledged [10–12]. It therefore remains unclear how automated scoring systems compare with expert human visual grading and what the potential impact is of CT-based AC. The current sub-analysis of the PACIFIC trial aims to compare the diagnostic accuracy of expert core laboratory reading of SPECT MPI against automated software package grading with and without CT-based AC, for the detection of obstructive CAD with FFR as a reference.

## Methods

### Patient population

The study population comprised 206 patients from the PACIFIC study (Comparison of Coronary CT Angiography, Myocardial Perfusion SPECT, PET, and Hybrid Imaging for Diagnosis of Ischemic Heart Disease: Prospective Cohort Study Using Fractional Flow Reserve to Determine Functional Severity of Coronary Stenoses; NCT01521468), who underwent ECG-gated SPECT/CT and invasive coronary angiography (ICA) with routine interrogation by FFR [13]. Enrolled patients were suspected of stable CAD with an intermediate pre-test likelihood and a normal left ventricular function. Exclusion criteria were a documented history of CAD, signs of prior myocardial infarction, contraindication to adenosine, atrial fibrillation, glomerular filtration rate < 45 mL∙min^−1^, and pregnancy.

### Image acquisition and reconstruction

Patients were instructed to refrain from intake of products containing caffeine or xanthine 24 h prior to the scans, next to at least 4 h of fasting. A 2-day stress-rest ^99m^Tc-tetrofosmin protocol was performed in all patients. During continuous infusion of adenosine (140 μg∙kg^−1^∙min^−1^), a weight-adjusted dose of 370 to 550 MBq ^99m^Tc-tetrofosmin was injected. The adenosine infusion was terminated 3 min after tracer injection. Following a delay of 60 min, ECG-gated stress SPECT images were acquired. Rest SPECT imaging was performed on the same day as ICA. Images were acquired on a dual-head hybrid SPECT/CT scanner (Symbia T2, Siemens Medical Solutions, Erlangen, Germany). Emission data were acquired using a parallel-hole, low energy, high resolution collimator with a 20% symmetric window centred at 140 keV, where the two detector heads were positioned at an angle of 90°. The camera heads performed a 180° rotation with, in total, 64 rotational steps of 40 s per projection. ECG-gating was performed with an electrocardiogram R-wave detector with acquisition of 8 frames per cardiac cycle. Images were reconstructed in both a static and gated manner. Characteristics of the embedded two-slice CT component were as follows: slice width 5.0 mm; pitch 1.5; 130 kV, 17 mA; rotation time 0.8 s. SPECT acquisition was followed immediately by a low-dose CT scan during normal breathing and without ECG-gating to correct for attenuation using 130 keV, 20 mAs, a computed tomography dose index of 2.2, and a dose length product of 40. CT images were reconstructed with a 128 × 128 matrix and a slice thickness of 5 mm.

### Visual analysis

Visual interpretation was performed by a core laboratory. A highly experienced observer (SRU, > 30 years of experience in nuclear cardiology) was blinded to other imaging and angiographic findings, but limited clinical information was available (patient's sex, age, body mass index, type of chest pain, and the presence of a left bundle branch block) because of the direct effects on scan interpretation. MPI images were interpreted based on a 17-segment model [14]. Each segment was scored using a 5-point scoring system (0, normal; 1, mildly decreased; 2, moderately decreased; 3, severely decreased; and 4, absence of segmental uptake). Summed rest scores (SRS), summed stress scores (SSS), and summed difference scores (SDS) were calculated from the segmental scores, with SSS ≥ 4 and SDS ≥ 2 considered abnormal [15, 16]. The expert reader was able to take into account additional information such as raw projections, ECG-gated LV functional information, as well as non-corrected (NC) and AC reconstructions in order to maximize recognition imaging artifacts. Visual interpretation was conclusively classified as normal or abnormal on a per patient basis.

### Automated analysis

Perfusion parameters were derived in an entirely automated fashion using commercially available software (Cedars-Sinai Quantitative Perfusion SPECT [QPS]) [2, 17, 18]. Each scoring parameter was derived from images with and without AC, representing both the extent and severity of myocardial hypoperfusion. These parameters comprise both the aforementioned scores based on the average defect severity per segment (SSS and SDS), as well as the pixel-wise total perfusion deficit (TPD) during stress (S-TPD) and the ischemic TPD (I-TPD), defined as the difference between stress and rest TPD [18]. SSS ≥ 4, SDS ≥ 2, S-TPD ≥ 5%, and I-TPD ≥ 3% were considered abnormal [15, 16, 19, 20].

Potential enhancement of the diagnostic performance of automated quantitative scoring was also explored. For this purpose, the total study database was consecutively divided into two subgroups. The optimization process comprised two components. First, an institutional normal database was created using data from the first subgroup, the derivation cohort (*n* = 103). The normal database was developed with SPECT images derived from patients with both normal angiographic findings as well as normal myocardial perfusion using [^15^O]H_2_O positron emission tomography (PET) imaging, which was additionally performed in the context of the PACIFIC trial [13]. By doing so, only SPECT images derived from patients without CAD were selected. The institutional database could then be generated within the commercially available software package. Second, optimal thresholds were obtained from the derivation cohort for each grading parameter with and without AC. Automated scoring was subsequently performed in the validation cohort (*n* = 103) with the use of the new normal database and optimized thresholds for abnormal scans.

### Invasive coronary angiography and fractional flow reserve

ICA imaging was performed using a standard protocol in at least two orthogonal directions per evaluated coronary artery segment. In order to induce epicardial coronary vasodilation, 0.2 mL of nitroglycerin was administered intracoronary ahead of contrast injection. All major coronary arteries were interrogated routinely by FFR, regardless of stenosis severity, except for occluded or subtotal lesions of more than 90%. FFR was measured using a 0.014-inch sensor tipped guide wire (Volcano Corporation, Rancho Cordova, CA, USA), which was introduced through a 5- or 6-F guiding catheter, calibrated and advanced into the coronary artery. Intracoronary (150 μg) or intravenous (140 μg∙kg^−1^∙min^−1^) adenosine infusion was used to induce maximal coronary hyperaemia. FFR was calculated as the ratio of mean distal intracoronary pressure, and mean arterial pressure. A coronary lesion was considered hemodynamically significant in case of FFR ≤ 0.80, or stenosis severity >90% obtained with quantitative coronary angiography (QCA) if FFR was missing. A stenosis with an FFR > 0.80, or a stenosis severity <30% (obtained with QCA) in the absence of FFR measurements, was not considered to be functionally relevant. Secondary analyses were performed using QCA stenosis severity as a reference with ≥70% stenosis considered obstructive. All images and FFR signals were interpreted by two experienced interventional cardiologists blinded to the SPECT result.

### Statistical analysis

Continuous variables were expressed as mean ± standard deviation, and categorical variables were expressed as percentages (%).The total estimate of agreement, defined as total cases where the tests agreed, was compared between automated and visual reads. Receiver operating characteristic (ROC) curves were performed to evaluate the ability of automated and visual scoring for predicting significant CAD. Optimal thresholds were established with the use of these ROC curves and the Youden index. The McNemar test was used to compare binary diagnostic performances of two assessments. For all analyses, *p* values <0.05 were considered statistically significant. Data were analyzed using SPSS Statistics version 20 (IBM Corporation, Armonk, NY, USA) and MedCalc version 10.3.0.0 Software (Mariakerke, Belgium).

## Results

**Fig. 1:**
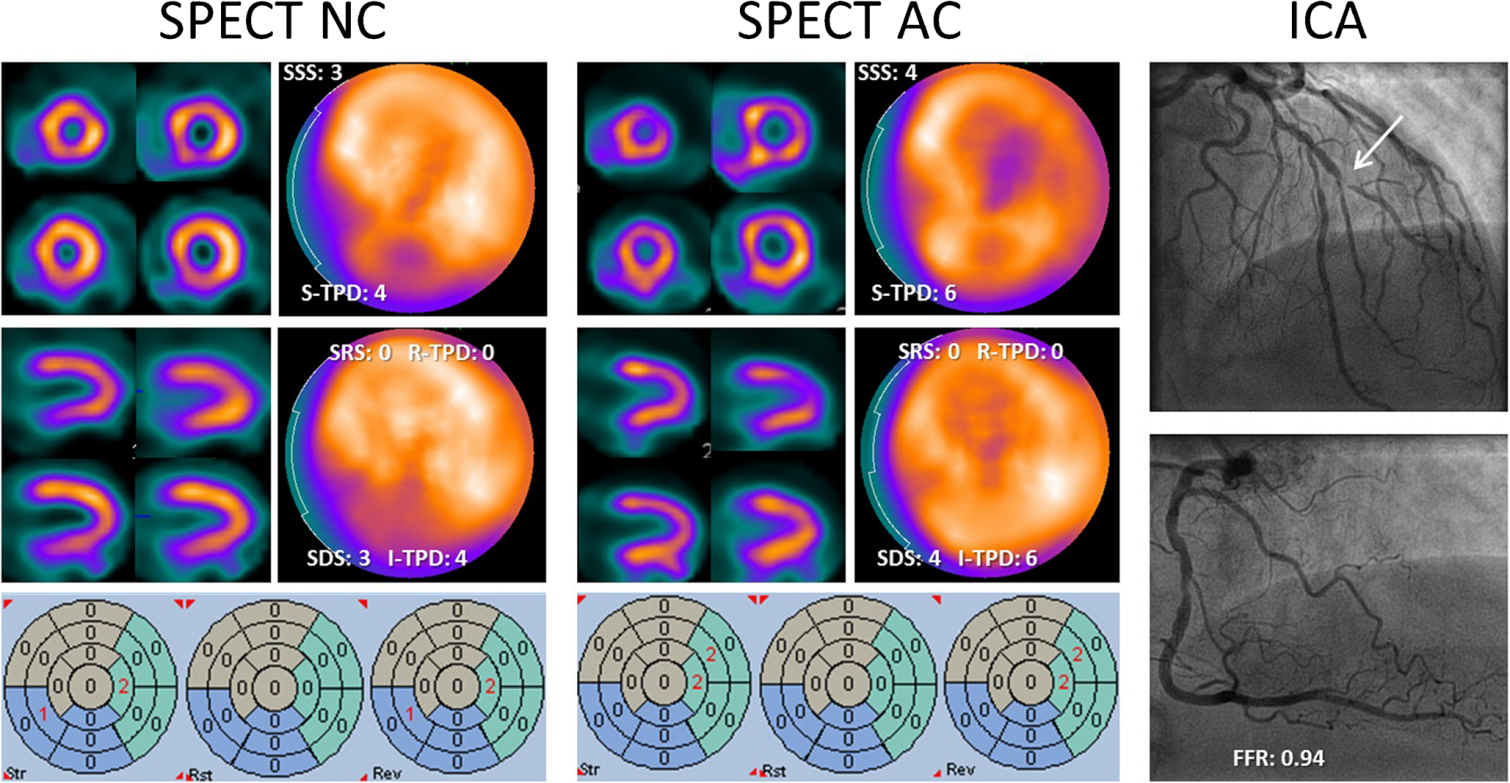
Representative SPECT images with and without AC, and invasive coronary angiography images of an 80-year-old male with typical angina. The left panel shows stress (upper row) and rest (lower row) images without AC. Only subtle perfusion reversibility can be observed in the anterior territory, whereas a fixed defect might be identified visually in the inferior territory. Automated grading revealed rather low scoring values, which were nonsignificant except for SDS and I-TPD. SPECT images with AC in the center panel display a slightly different perfusion pattern with more pronounced reversibility in the anterolateral segments, whereas the inferior wall is corrected into normal perfusion. Automated grading now clearly indicates ischemia in the anterior region only, instead of possible ischemia anterior and inferior. A sub-totally occluded diagonal branch but non-significant stenosis in the RCA on angiographic images (right panel) confirm the SPECT findings. AC = attenuation correction; FFR = fractional flow reserve; I-TPD = ischemic total perfusion deficit; NC = non-corrected; SDS = summed difference score; SRS = summed rest score; SSS = summed stress score; R-TPD = rest total perfusion deficit; S-TPD = stress total perfusion deficit

Baseline characteristics of the study population are listed in Table 1. In brief, the mean age was 58.2 ± 8.7 years, 64% were male, and 92 (45%) patients were found to have significant CAD as defined by invasive coronary angiography with an FFR ≤ 0.80. In total, FFR was measured in 548 vessels, but not in 61 due to complete (*n* = 24) and sub-total (*n* = 34) occlusions (deemed hemodynamically significant), or severe coronary tortuosity (*n* = 3, no stenosis ≥30%, considered not hemodynamically significant CAD). Mean radiation dose for SPECT was 4.89 ± 0.71 mSv without low-dose CT and 6.01 ± 0.71 mSv with low-dose CT for attenuation correction. Additionally, visual expert analysis resulted in 59 (29%) abnormal SPECT studies. A case example is shown in Fig. 1.

**Table 1:** Patient baseline characteristics

Parameter	Total group (*n* = 206)	Derivation group (*n* = 103)	Validation group (*n* = 103)	*P* for subgroups
*Demographics*				
Age, years	58.2 ± 8.7	57.8 ± 9.3	58.6 ± 8.1	0.501
Male	131 (64%)	64 (62%)	67 (65%)	0.666
BMI	27.0 ± 3.7	27.2 ± 3.9	26.9 ± 3.6	0.547
LBBB	6 (3%)	3 (3%)	3 (3%)	0.990
*CAD risk factors*				
Hypertension	95 (46%)	49 (48%)	46 (45%)	0.677
Hyperlipidemia	83 (40%)	41 (40%)	42 (41%)	0.888
Diabetes	33 (16%)	16 (16%)	17 (17%)	0.850
Current smoker	40 (19%)	19 (18%)	21 (20%)	0.726
Smoking history	99 (48%)	49 (48%)	50 (49%)	0.890
Family history of CAD	105 (51%)	49 (48%)	56 (54%)	0.332
Significant CAD	92 (45%)	44 (43%)	48 (47%)	0.577

Values are mean ± SD or no. (%)

BMI = body mass index; CAD = coronary artery disease; LBBB = left bundle branch block

### Diagnostic performance of visual and standard automated assessment

Table 2 shows diagnostic performance in terms of sensitivity, specificity, and diagnostic accuracy of expert visual reading and multiple automated measurements for the detection of hemodynamically significant CAD. These parameters were scored using normal scan databases incorporated in the commercially available software and accepted thresholds of abnormality. Among all automatically assessed scores, only SDS with and without AC (86.5% and 80.0%, respectively, *p* < 0.001 for both) showed a significantly higher sensitivity than expert reading (56.5%). Sensitivity of the other parameters, including SSS, S-TPD and I-TPD, did not statistically differ from visual analysis, even though a trend was visible in favor of automated analysis. In contrast, specificity of expert reading (93.9%) was significantly higher than that of each of the automatically derived scores. In terms of diagnostic accuracy, automated assessment performed more poorly than visual reading (77.2%), except for SSS AC (69.8%, *p* = 0.063) and S-TPD AC (71.2%, *p* = 0.134).

**Table 2:** Diagnostic performance of expert visual analysis and automated analysis using standard software for the detection of coronary artery disease (*n* = 206)

	Sensitivity	Difference with expert	Specificity	Difference with expert	Diagnostic accuracy	Difference with expert
Expert	56.5 (45.8–66.8)		93.9 (87.8–97.5)		77.2 (70.8–82.7)	
SSS NC	66.3 (55.7–75.8)	*P* = 0.108	68.4 (59.1–76.8)	*P* < 0.001*	67.5(60.6–73.8)	*P* = 0.012*
SSS AC	67.0 (56.4–76.5)	*P* = 0.064	71.9 (62.7–79.9)	*P* < 0.001*	69.8 (63.0–76.0)	*P* = 0.063
SDS NC	80.0 (70.3–87.7)	*P* < 0.001*	50.0 (40.5–59.5)	*P* < 0.001*	63.2 (56.2–69.9)	*P* = 0.003*
SDS AC	86.5 (77.6–92.8)	*P* < 0.001*	49.1 (39.6–58.7)	*P* < 0.001*	65.5 (58.5–72.0)	*P* = 0.023*
S-TPD NC	62.0 (51.2–71.9)	*P* = 0.405	73.7 (64.6–81.5)	*P* < 0.001*	68.4 (61.6–74.7)	*P* = 0.020*
S-TPD AC	64.8 (54.1–74.6)	*P* = 0.152	76.3 (67.4–83.8)	*P* < 0.001*	71.2 (64.5–77.3)	*P* = 0.134
I-TPD NC	65.6 (54.8–75.3)	*P* = 0.108	69.9 (60.6–78.2)	*P* < 0.001*	68.0 (61.1–74.3)	*P* = 0.027*
I-TPD AC	65.9 (55.3–75.6)	*P* = 0.108	65.8 (56.3–74.4)	*P* < 0.001*	65.8 (58.9–72.3)	*P* = 0.005*

Values are no. (95% confidence interval)

AC = attenuation correction; I-TPD = ischemic total perfusion deficit; NC = non-corrected; SDS = summed difference score; SSS = summed stress score; S-TPD = stress total perfusion deficit. *Indicating a significant difference with expert visual analysis (*P* < 0.05)

### Optimizing automated assessment

**Fig. 2:**
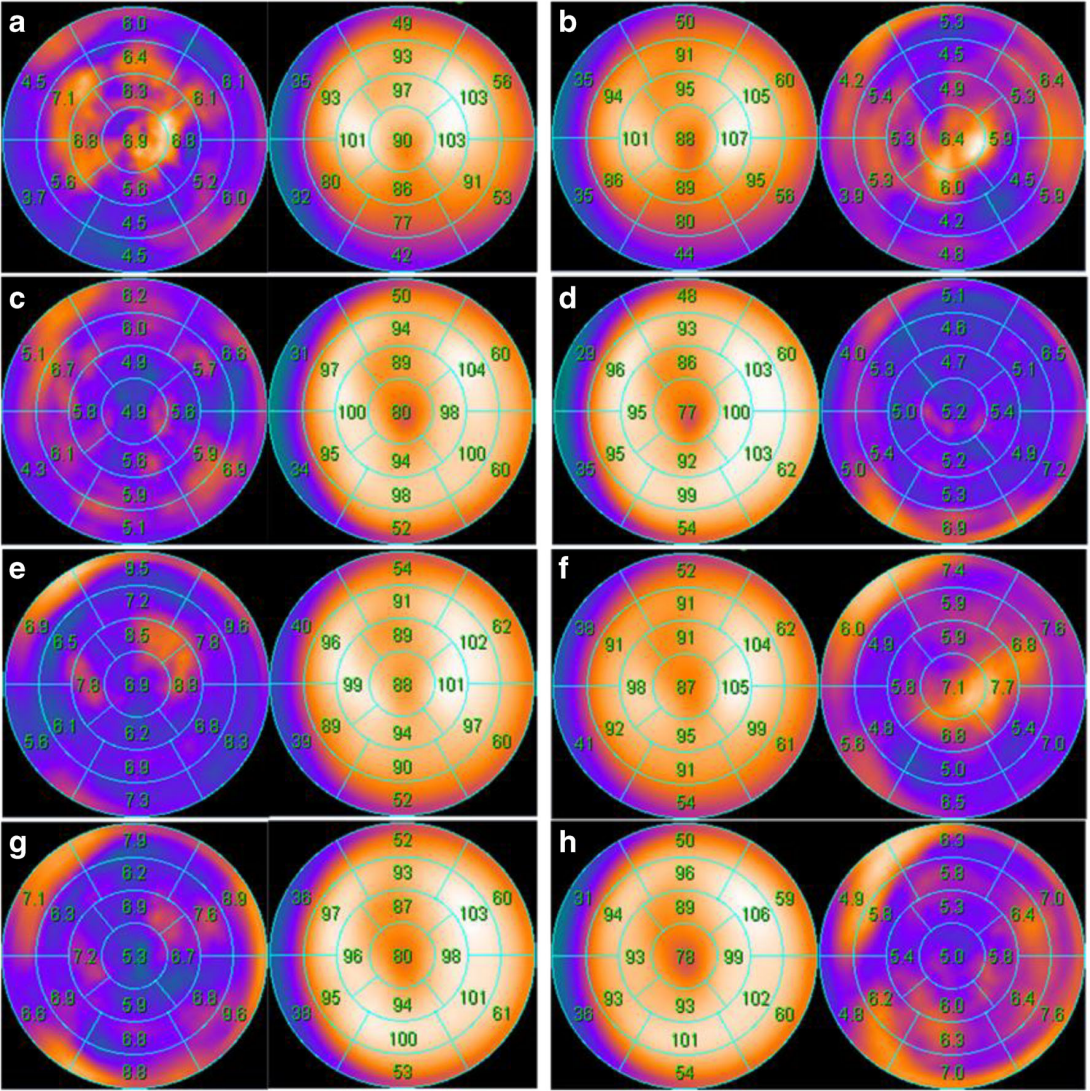
Average polar maps for male (**a**–**d**) and female (**e**–**h**) from the newly derived institutional normal database in the left column and from the vendor-supplied normal database in the right column. Polar maps **a**, **b**, **e**, and **f** are created from non-attenuation corrected images, whereas polar maps **c**, **d**, **g**, and **h** are derived from attenuation corrected images

**Fig. 3:**
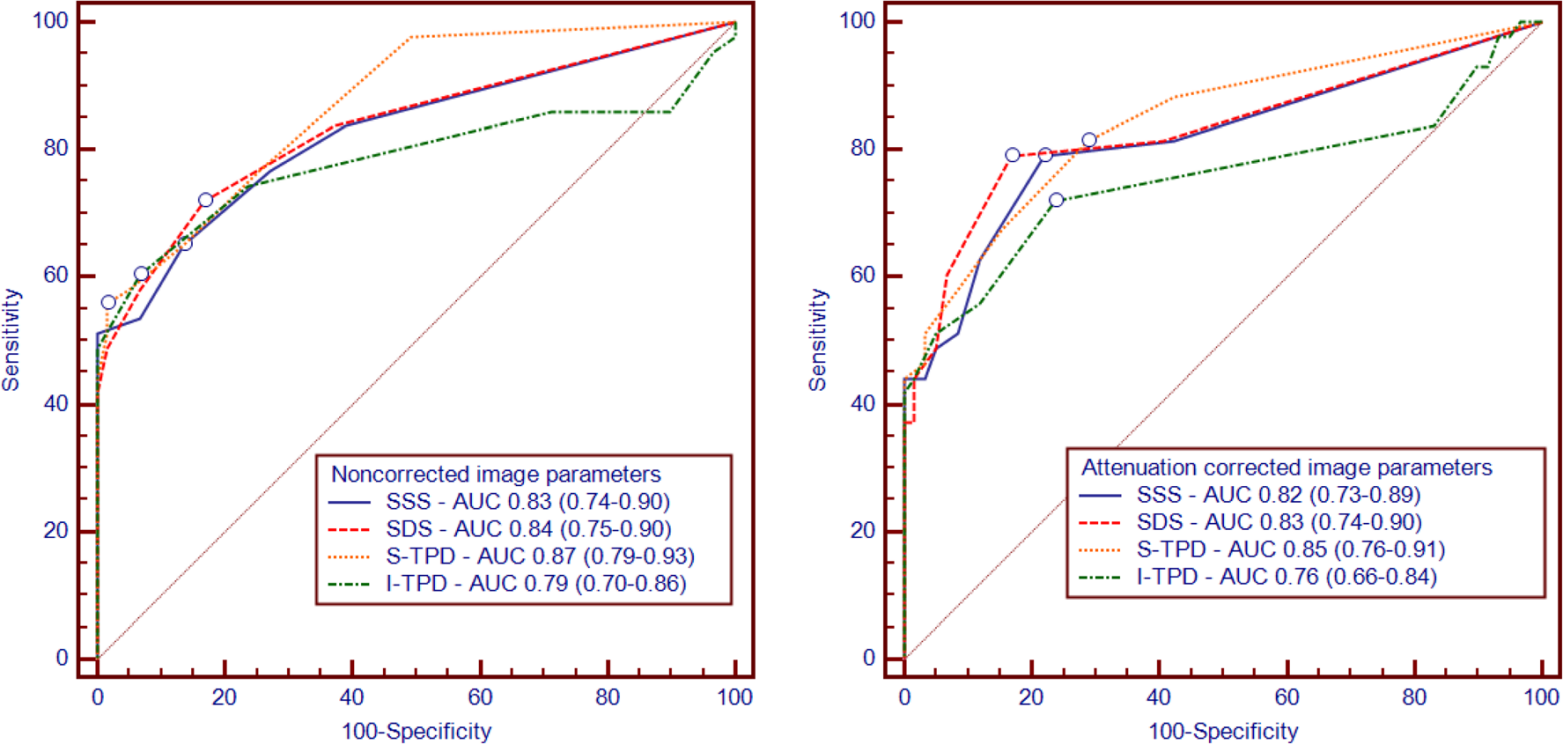
Receiver operating characteristic curves for predicting significant coronary artery disease, defined by an FFR ≤ 0.80, in the derivation cohort using the new normal databases for NC (left panel) and AC (right panel) automated parameters (SSS, SDS, S-TPD, and I-TPD). The lines represent prognostic sensitivity and false positive rates at increasing threshold values. Areas under the curves and 95% confidence intervals were calculated for each parameter. Threshold values with the highest Youden index for each curve are marked with open dots. Abbreviations as in Fig. 1

After dividing the total group of patients into derivation and validation cohorts (*n* = 103 for both), 51 normal SPECT images (including 30 female patients images) were used for the development of a new institutional database. As listed in Table 1, there were no differences in baseline characteristics between the derivation and validation cohorts. Figure 2 shows the average polar maps for the normal database, incorporated in the software package by the vendor, next to the average polar maps derived from the newly generated institutional normal database. Cases selected for the new normal database (normal FFR and normal PET perfusion) showed an SSS = 0 in 7 (14%) cases and an abnormal SSS ≥ 4 in 15 (29%) with the use of the original database. Mean SSS values decreased implementing the institutional database towards SSS = 0 in 34 (67%) cases and SSS ≥ 4 in 1 (2%) case. Based on the derivation cohort and the new normal databases, optimal thresholds were set at SSS ≥ 3, SDS ≥ 2, S-TPD ≥ 5 and I-TPD ≥ 2 for images without AC (AUC: 0.83, 0.84, 0.87, and 0.79, respectively), and at SSS ≥ 2, SDS ≥ 2, S-TPD ≥ 2 and I-TPD ≥ 1 for images with AC (AUC 0.82, 0.83, 0.85, and 0.76, respectively; Fig. 3).

### Diagnostic performance of optimized automated assessment

Table 3 presents the diagnostic performance of expert visual reading and automated assessment in the validation cohort using the institutional database and optimal thresholds. The sensitivities for NC images were consistently low and did not significantly differ from visual reads. Automated scoring with AC images provided higher sensitivities, although this difference was significant only for S-TPD and I-TPD (*p* = 0.001 and *p* = 0.008, respectively). In contrast, the visually obtained high specificity remained significantly different from all automated AC scores, whereas NC images did not show a significant difference. Consequently, diagnostic accuracy for each automated assessment did not significantly differ from expert visual analysis. Explicitly, the highest accuracies were found for SSS AC (72.5%), SDS AC (72.0%), and S-TPD AC (73.5%) and paralleled expert analysis (73.8%).

**Table 3:** Diagnostic performance of expert visual analysis and automated analysis using optimized software with a new normal database and thresholds, for the detection of coronary artery disease in the validation cohort (*n* = 103)

	Sensitivity	Difference with expert	Specificity	Difference with expert	Diagnostic accuracy	Difference with expert
Expert	52.1 (37.2–66.7)		92.7 (82.4–98.0)		73.8 (64.2–82.0)	
SSS NC	52.1 (37.2–66.7)	*P* = 1.000	83.6 (71.2–92.2)	*P* = 0.227	68.9 (59.1–77.7)	*P* = 0.383
SSS AC	68.1 (52.9–80.9)	*P* = 0.077	76.4 (63.0–86.8)	*P* = 0.035*	72.5 (62.8–80.9)	*P* = 1.000
SDS NC	50.0 (34.9–65.1)	*P* = 1.000	80.0 (67.0–89.6)	*P* = 0.092	66.3 (56.3–75.4)	*P* = 0.210
SDS AC	66.7 (51.1–80.0)	*P* = 0.077	76.4 (63.0–86.8)	*P* = 0.035*	72.0 (62.1–80.5)	*P* = 1.000
S-TPD NC	45.8 (31.4–60.8)	*P* = 0.508	92.7 (82.4–98.0)	*P* = 1.000	70.9 (61.1–79.4)	*P* = 0.607
S-TPD AC	83.0 (69.2–92.4)	*P* = 0.001*	65.5 (51.4–77.8)	*P* < 0.001*	73.5 (63.9–81.8)	*P* = 1.000
I-TPD NC	46.8 (32.1–61.9)	*P* = 0.774	78.2 (65.0–88.2)	*P* = 0.057	63.7 (53.6–73.0)	*P* = 0.076
I-TPD AC	76.6 (62.0–87.7)	*P* = 0.008*	60.0 (45.9–73.0)	*P* < 0.001*	67.6 (57.7–76.6)	*P* = 0.418

Values are no. (95% confidence interval)

Abbreviations as in Table 2. * Indicates a significant difference with expert visual analysis (*P* < 0.05)

### Diagnostic performance using angiographic stenosis severity as a reference

Using QCA instead of FFR as a reference, the performance of the expert reader was enhanced, mainly due to a higher sensitivity (online Tables 1 and 2). In general, sensitivity also increased for automated analysis while specificity generally decreased, yielding a heterogeneous change in performance in terms of diagnostic accuracy. Nevertheless, diagnostic accuracy was significantly lower for all automated scoring variables with the use of standard software (online table 1). After the software optimization, NC parameters were comparable to visual reading with regard to diagnostic accuracy, except for I-TPD which was significantly lower. Among the AC parameters, SSS and SDS showed comparable accuracy, whereas S-TPD and I-TPD revealed significantly lower accuracy (online table 2). AUCs of the automated parameters did not significantly differ, but in general, a trend was observed for the numerical smaller AUCs for I-TPD with and without AC (online Figs. 1 and 2).

## Discussion

At present, the standard clinical practice for evaluation of SPECT images is visual assessment, which depends on the skills of the reader and which is rather subjective. The present study demonstrates that, in general, automated analysis has a lower diagnostic accuracy than visual analysis, predominantly instigated by a lower specificity. After the introduction of an institutional normal database and optimization of thresholds, however, diagnostic accuracy of the automated analysis increased and no longer differed from expert visual reading. A novelty of this prospective study comparing expert visual analysis and automated computer analysis is that every patient underwent invasive coronary angiography with routine FFR measurements as a reference standard.

### Visual versus automated assessment of SPECT perfusion

An accurate assessment of the extent and severity of hypoperfused myocardium with SPECT is important for diagnostic and prognostic purposes [17, 19, 21], but remains subjective when determined visually. Therefore, software tools for automatic quantification have been developed, and subsequently it has been shown that these are more reproducible than visual assessment, even if the latter is performed by highly experienced readers [9, 22]. Arsanjani et al. showed that diagnostic performance of automated analysis did not significantly differ from that of visual assessment [7]. Although these results were promising, the study was hampered by the fact that a significant portion of the study population was expected to be free of CAD based on low risk profiles without confirmation through ICA. Another limitation of several diagnostic studies is that when study patients did undergo ICA, visual estimation of stenosis severity was used as the reference standard, but frequent disagreement between angiographic visual stenosis severity and functional severity is increasingly recognized [11]. As a sub-analysis from the PACIFIC trial, the present study was not hampered by these limitations, as all subjects underwent invasive coronary angiography with routine FFR measurements [13].

The present results from automated analysis using standard software and thresholds, in general, revealed a lower diagnostic accuracy than visual expert reading except for SSS and S-TPD with AC. Furthermore, automated analysis showed higher sensitivity, but lower specificity, than visual reading. This implies that when automated analysis is used for diagnostic purposes, AC is warranted and stress-only protocols are sufficient. Nevertheless, it should be realized that these results hold true only for this particular study population of patients with a normal left ventricular function without prior history of CAD or myocardial infarction. In addition, the slightly higher sensitivity of automated analysis in trade off of a lower specificity could be more favorable as the number of ‘unnecessary’ invasive angiograms might outweigh missing obstructive CAD due to a false negative SPECT. Of note, it seems important to see that the use of FFR instead of the more traditionally used QCA as a reference changed the results to some extent (online table 1). One explanation for the improved performance of visual reading using QCA as a reference, might be the reader’s experience and prior feedback based on QCA rather than FFR (i.e. the readers ‘internal normal database’).

### Normal database

The technique for automated quantification of myocardial perfusion relies on the analysis of tracer distribution within one patient, which then is compared with a database of normal perfusion scans. This so called normal database is usually based on perfusion images from the USA, since most software packages are developed there. Given possible differences in patient habitus and imaging protocols (including tracer doses and scanning settings), this database may not be optimal for other regions [23]. An interesting study from Nakajima et al. showed a significant diagnostic improvement with the use of a region specific normal database in Japan [24]. In contrast, a similar study with a French population did not show a clear benefit compared with the normal database supplied by the vendor [25]. The present study demonstrates the feasibility of creating an institution normal database with only a limited number of normal perfusion studies. However, reviewing Fig. 2 reveals merely minor differences for average polar maps between the present institutional database and the vendor supplied database. Nonetheless, the normalcy rate improved from 71% to 98% and the majority of diagnostic accuracies for automated analyses in the validation cohort directly increased after implementing the institutional database (online Fig. 2). The most pronounced differences (according to average segmental counts) seem to be located in the anterior and basal inferior regions for NC images, probably as a result of attenuation differences due to surrounding soft tissue, such as breast and abdomen. Of interest, the normal tracer distribution of AC images, on average segmental counts maps and standard deviation maps, appears to be very similar for both gender averages as well as for vendor and institution databases. This suggests the possibility to easily exchange AC image normal databases [26]. A limitation of common normal databases is the typical use of SPECT images obtained from patients with a low pretest likelihood for CAD, who did not undergo ICA to confirm. Even though differences might be small, the current database is unique because normal perfusion was guaranteed through the confirmation with ICA and [^15^O]H_2_O PET imaging in prospectively enrolled patients.

### Optimization of automated analysis

The main purpose of generating an institutional normal database was to improve performance of the automated analysis. Based on the derivation cohort, optimal thresholds were slightly lower than the traditional cut-off values, particularly for AC thresholds. The diagnostic accuracy of the newly set thresholds was higher for attenuation corrected images, confirming the benefit of attenuation correction when automated analysis is used. Although diagnostic accuracy was consistently higher with AC, the question remains whether this justifies the additional radiation burden for patients, or costs and time for imaging laboratories. Furthermore, derivation cohort AC images did not show an improved accuracy when using AUCs, which provide a comprehensive and likely a more adequate evaluation of the diagnostic performance than the dichotomized accuracy (Fig. 3). The validation cohort revealed that implementation of a normal database and optimized thresholds now resulted in an equivalent diagnostic accuracy of automated analysis as compared with visual expert grading for all investigated parameters (SSS, SDS, S-TPD and I-TPD). Using QCA as a reference instead of FFR, diagnostic accuracies of most optimized automated analysis parameters were not significantly different from visual reading (online table 2). In general, however, these numerical differences were somewhat more pronounced than for the comparisons referenced by FFR. The fact that the institutional normal database was created with the use of FFR rather than QCA might have played a role.

Recently, several developments have been implemented clinically in order to improve diagnostic performance of conventional SPECT imaging. For instance, ECG-gated acquisitions provide additional diagnostic and prognostic information such as end-diastolic volume, ejection fraction and transient ischemic dilatation [27–29]. These functional parameters were not included in the present automated analysis, but hold great potential for further improvement of the automatic SPECT interpretation, for example using machine learning programs [30].

## Limitations

Some limitations should be noted in the context of this study. First, the study population consisted of a total of 206 patients with two test groups of 103 patients, which might be enough to perform comparisons, but may be rather small to detect significant differences. Accordingly, also, the newly derived normal database is relatively small. Despite Slomka et al. [31] having recommended 20–40 images to create a reliable normal database, an appealing study from Tragardh et al. [32] demonstrated an improved accuracy with an increasing database size, up to 100 images. Furthermore, present analyses were performed with one specific scanning protocol and study population and compared with a single expert visual reader. Current findings would therefore not be interchangeable with other institutions using different imaging protocols, other subgroups of patients, and other visual reviewers. Finally, it has to be acknowledged that diagnostic accuracy results depend on the prevalence of disease.

### Conclusion

Visual analysis of SPECT imaging slightly outperforms automated analysis with standard software in the detection of FFR-defined significant CAD. After optimization with an institutional normal database and thresholds, however, diagnostic accuracy of automated analysis equalled expert visual analysis without the need for comprehensive reading experience. Therefore, automatic assessment has the potential to simplify the diagnostic process using SPECT, particularly in conjunction with CT-based AC.

## Electronic supplementary material

ESM 1 (DOCX 582 kb)

## References

[1] McNulty EJ, Hung YY, Almers LM, Go AS, Yeh RW (2014). Population trends from 2000-2011 in nuclear myocardial perfusion imaging use. JAMA.

[2] Germano G, Kavanagh PB, Slomka PJ, Van Kriekinge SD, Pollard G, Berman DS (2007). Quantitation in gated perfusion SPECT imaging: the Cedars-Sinai approach. J Nucl Cardiol.

[3] Ficaro EP, Lee BC, Kritzman JN, Corbett JR (2007). Corridor4DM: the Michigan method for quantitative nuclear cardiology. J Nucl Cardiol.

[4] Garcia EV, Faber TL, Cooke CD, Folks RD, Chen J, Santana C (2007). The increasing role of quantification in clinical nuclear cardiology: the Emory approach. J Nucl Cardiol.

[5] Duvall WL, Slomka PJ, Gerlach JR, Sweeny JM, Baber U, Croft LB, Guma KA, George T, Henzlova MJ (2013). High-efficiency SPECT MPI: comparison of automated quantification, visual interpretation, and coronary angiography. J Nucl Cardiol.

[6] Xu Y, Fish M, Gerlach J, Lemley M, Berman DS, Germano G, Slomka PJ (2010). Combined quantitative analysis of attenuation corrected and non-corrected myocardial perfusion SPECT: method development and clinical validation. J Nucl Cardiol.

[7] Arsanjani R, Xu Y, Hayes SW, Fish M, Lemley M, Gerlach J, Dorbala S, Berman DS, Germano G, Slomka P (2013). Comparison of fully automated computer analysis and visual scoring for detection of coronary artery disease from myocardial perfusion SPECT in a large population. J Nucl Med.

[8] Holly TA, Abbott BG, Al-Mallah M, Calnon DA, Cohen MC, DiFilippo FP, Ficaro EP, Freeman MR, Hendel RC, Jain D, Leonard SM, Nichols KJ, Polk DM, Soman P (2010). Single photon-emission computed tomography. J Nucl Cardiol.

[9] Berman DS, Kang X, Gransar H, Gerlach J, Friedman JD, Hayes SW, Thomson LE, Hachamovitch R, Shaw LJ, Slomka PJ, Yang LD, Germano G (2009). Quantitative assessment of myocardial perfusion abnormality on SPECT myocardial perfusion imaging is more reproducible than expert visual analysis. J Nucl Cardiol.

[10] Tonino PA, De BB, Pijls NH, Siebert U, Ikeno F, Veer M, Klauss V, Manoharan G, Engstrom T, Oldroyd KG, Ver Lee PN, Maccarthy PA, Fearon WF (2009). Fractional flow reserve versus angiography for guiding percutaneous coronary intervention. N Engl J Med.

[11] Tonino PA, Fearon WF, De BB, Oldroyd KG, Leesar MA, Ver Lee PN, Maccarthy PA, Van't Veer M, Pijls NH (2010). Angiographic versus functional severity of coronary artery stenoses in the FAME study fractional flow reserve versus angiography in multivessel evaluation. J Am Coll Cardiol.

[12] Johnson NP, Toth GG, Lai D, Zhu H, Acar G, Agostoni P, Appelman Y, Arslan F, Barbato E, Chen SL, Di SL, Dominguez-Franco AJ, Dupouy P, Esen AM, Esen OB, Hamilos M, Iwasaki K, Jensen LO, Jimenez-Navarro MF, Katritsis DG, Kocaman SA, Koo BK, Lopez-Palop R, Lorin JD, Miller LH, Muller O, Nam CW, Oud N, Puymirat E, Rieber J, Rioufol G, Rodes-Cabau J, Sedlis SP, Takeishi Y, Tonino PA, Van BE VE, Werner GS, Fearon WF, Pijls NH, De BB, Gould KL (2014). Prognostic value of fractional flow reserve: linking physiologic severity to clinical outcomes. J Am Coll Cardiol.

[13] Danad I, Raijmakers PG, Driessen RS, Leipsic J, Raju R, Naoum C, Knuuti J, Maki M, Underwood S, Min JK, Elmore K, Stuijfzand WJ, van Royen N, Tulevski II, Somsen GA, Huisman MC, van Lingen A, Heymans MW, van de Ven PM, van Kuijk CC, Lammertsma AA, van Rossum AC, Knaapen P: Comparison of coronary CT angiography, SPECT, PET, and Hybrid Imaging for Diagnosis of Ischemic Heart Disease Determined by Fractional Flow Reserve. JAMA Cardiol 2017. 10.1001/jamacardio.2017.2471 PMC5710451 28813561

[14] Cerqueira MD, Weissman NJ, Dilsizian V, Jacobs AK, Kaul S, Laskey WK, Pennell DJ, Rumberger JA, Ryan T, Verani MS: Standardized myocardial segmentation and nomenclature for tomographic imaging of the heart. A statement for healthcare professionals from the cardiac imaging Committee of the Council on clinical cardiology of the American Heart Association. Circulation 2002;105:539-542. 10.1161/hc0402.102975 11815441

[15] van Werkhoven JM, Schuijf JD, Gaemperli O, Jukema JW, Boersma E, Wijns W, Stolzmann P, Alkadhi H, Valenta I, Stokkel MP (2009). Kroft LJ, de RA, Pundziute G, Scholte a, van der Wall EE, Kaufmann PA, Bax JJ: prognostic value of multislice computed tomography and gated single-photon emission computed tomography in patients with suspected coronary artery disease. J Am Coll Cardiol.

[16] Neglia D, Rovai D, Caselli C, Pietila M, Teresinska A, Aguade-Bruix S, Pizzi MN, Todiere G, Gimelli A, Schroeder S, Drosch T, Poddighe R, Casolo G, Anagnostopoulos C, Pugliese F, Rouzet F, Le GD, Cappelli F, Valente S, Gensini GF, Zawaideh C, Capitanio S, Sambuceti G, Marsico F, Perrone FP, Fernandez-Golfin C, Rincon LM, Graner FP, de Graaf MA, Fiechter M, Stehli J, Gaemperli O, Reyes E, Nkomo S, Maki M, Lorenzoni V, Turchetti G, Carpeggiani C, Marinelli M, Puzzuoli S, Mangione M, Marcheschi P, Mariani F, Giannessi D, Nekolla S, Lombardi M, Sicari R, Scholte AJ, Zamorano JL, Kaufmann PA, Underwood SR, Knuuti J: Detection of significant coronary artery disease by noninvasive anatomical and functional imaging. Circ Cardiovasc Imaging 2015;8. 10.1161/CIRCIMAGING.114.002179 25711274

[17] Germano G, Kavanagh PB, Waechter P, Areeda J, Van KS, Sharir T, Lewin HC, Berman DS (2000). A new algorithm for the quantitation of myocardial perfusion SPECT. I: technical principles and reproducibility. J Nucl Med.

[18] Slomka PJ, Nishina H, Berman DS, Akincioglu C, Abidov A, Friedman JD, Hayes SW, Germano G (2005). Automated quantification of myocardial perfusion SPECT using simplified normal limits. J Nucl Cardiol.

[19] Shaw LJ, Berman DS, Maron DJ, Mancini GB, Hayes SW, Hartigan PM, Weintraub WS, O'Rourke RA, Dada M, Spertus JA, Chaitman BR, Friedman J, Slomka P, Heller GV, Germano G, Gosselin G, Berger P, Kostuk WJ, Schwartz RG, Knudtson M, Veledar E, Bates ER, McCallister B, Teo KK, Boden WE (2008). Optimal medical therapy with or without percutaneous coronary intervention to reduce ischemic burden: results from the Clinical Outcomes Utilizing Revascularization and Aggressive Drug Evaluation (COURAGE) trial nuclear substudy. Circulation.

[20] Prasad M, Slomka PJ, Fish M, Kavanagh P, Gerlach J, Hayes S, Berman DS, Germano G (2010). Improved quantification and normal limits for myocardial perfusion stress-rest change. J Nucl Med.

[21] Hachamovitch R, Rozanski A, Shaw LJ, Stone GW, Thomson LE, Friedman JD, Hayes SW, Cohen I, Germano G, Berman DS (2011). Impact of ischaemia and scar on the therapeutic benefit derived from myocardial revascularization vs. medical therapy among patients undergoing stress-rest myocardial perfusion scintigraphy. Eur Heart J.

[22] Xu Y, Hayes S, Ali I, Ruddy TD, Wells RG, Berman DS, Germano G, Slomka PJ (2010). Automatic and visual reproducibility of perfusion and function measures for myocardial perfusion SPECT. J Nucl Cardiol.

[23] Nakajima K, Okuda K, Kawano M, Matsuo S, Slomka P, Germano G, Kinuya S (2009). The importance of population-specific normal database for quantification of myocardial ischemia: comparison between Japanese 360 and 180-degree databases and a US database. J Nucl Cardiol.

[24] Yoda S, Nakanishi K, Tano A, Hori Y, Suzuki Y, Matsumoto N, Hirayama A (2013). Diagnostic value of automated quantification of nuclear cardiology in Japanese patients with single vessel coronary artery disease: comparison between Japanese and American normal databases. J Cardiol.

[25] Gregoire B, Pina-Jomir G, Bontemps L, Janier M, Scheiber C. The value of local normal limits in quantitative Thallium-201 CZT MPI SPECT. J Nucl Cardiol. 2016. 10.1007/s12350-016-0430-6 26936035

[26] Tragardh E, Sjostrand K, Jakobsson D, Edenbrandt L (2011). Small average differences in attenuation corrected images between men and women in myocardial perfusion scintigraphy: a novel normal stress database. BMC Med Imaging.

[27] Karimi-Ashtiani S, Arsanjani R, Fish M, Kavanagh P, Germano G, Berman D, Slomka P (2012). Direct quantification of left ventricular motion and thickening changes using rest-stress myocardial perfusion SPECT. J Nucl Med.

[28] Abidov A, Bax JJ, Hayes SW, Hachamovitch R, Cohen I, Gerlach J, Kang X, Friedman JD, Germano G, Berman DS (2003). Transient ischemic dilation ratio of the left ventricle is a significant predictor of future cardiac events in patients with otherwise normal myocardial perfusion SPECT. J Am Coll Cardiol.

[29] Genovesi D, Giorgetti A, Gimelli A, Kusch A, D’Aragona TI, Casagranda M, Cannizzaro G, Giubbini R, Bertagna F, Fagioli G, Rossi M, Romeo A, Bertolaccini P, Bonini R, Marzullo P (2011). Impact of attenuation correction and gated acquisition in SPECT myocardial perfusion imaging: results of the multicentre SPAG (SPECT attenuation correction vs gated) study. Eur J Nucl Med Mol Imaging.

[30] Arsanjani R, Dey D, Khachatryan T, Shalev A, Hayes SW, Fish M, Nakanishi R, Germano G, Berman DS, Slomka P (2015). Prediction of revascularization after myocardial perfusion SPECT by machine learning in a large population. J Nucl Cardiol.

[31] Slomka P, Xu Y, Berman D, Germano G (2012). Quantitative analysis of perfusion studies: strengths and pitfalls. J Nucl Cardiol.

[32] Tragardh E, Sjostrand K, Edenbrandt L (2012). Normal stress databases in myocardial perfusion scintigraphy--how many subjects do you need?. Clin Physiol Funct Imaging.

