# Supplementary material for: Automated SPECT analysis compared with expert visual scoring for the detection of FFR-defined coronary artery disease
Source: Eur J Nucl Med Mol Imaging. 2018 Feb 22;45(7):1091–100. doi: 10.1007/s00259-018-3951-1 (PMC5954003; doi:10.1007/s00259-018-3951-1)

**Supplemental tables and figures**

**Supplemental table 1:** Diagnostic performance of expert visual analysis and automated analysis using standard software for the detection of coronary artery disease as defined by stenosis ≥ 70% (n = 206).

**Supplemental table 2:** Diagnostic performance of expert visual analysis and automated analysis using optimized software, for the detection of coronary artery disease as defined by stenosis ≥ 70% in the validation cohort (n = 103).

**Supplemental figure 1: Standard software ROC curves for ≥70% stenosis.** Receiver operating characteristic curves for predicting obstructive coronary artery disease, defined by stenosis severity ≥ 70%, in the total study cohort using standard software for NC (left panel) and AC (right panel) automated parameters (SSS, SDS, S-TPD, and I-TPD). The lines represent prognostic sensitivity and false positive rates at increasing threshold values. Areas under the curves and 95% confidence intervals were calculated for each parameter. Threshold values with the highest Youden index for each curve are marked with open dots.

AC = attenuation correction; I-TPD = ischemic total perfusion deficit; NC = noncorrected; ROC = receiver operating characteristic; SDS = summed difference score; SSS = summed stress score; S-TPD = stress total perfusion deficit

**Supplemental figure 2: Optimized software ROC curves for ≥70% stenosis in validation cohort.** Receiver operating characteristic curves for predicting obstructive coronary artery disease, defined by stenosis severity ≥ 70%, in the validation cohort using the new normal databases for NC (left panel) and AC (right panel) automated parameters (SSS, SDS, S-TPD, and I-TPD). The lines represent prognostic sensitivity and false positive rates at increasing threshold values. Areas under the curves and 95% confidence intervals were calculated for each parameter. Threshold values with the highest Youden index for each curve are marked with open dots.

Abbreviations as in figure 1.

**Supplemental figure 3: Diagnostic performance in the validation cohort before and after optimization.** Diagnostic performance of automated analysis in the validation cohort (n = 103) before (1) and after (2) implementation of institutional normal database for noncorrected (A) and attenuation corrected (B) images.

Abbreviations as in figure 1.

**Supplemental table 1:** Diagnostic performance of expert visual analysis and automated analysis using standard software for the detection of coronary artery disease as defined by stenosis ≥ 70% (n = 206).

|  | Sensitivity | Difference with expert | Specificity | Difference with expert | Diagnostic accuracy | Difference with expert |
| --- | --- | --- | --- | --- | --- | --- |
| Expert | 70.0  (56.8 – 81.2) |  | 88.4  (82.0 – 93.1) |  | 83.0  (77.2 – 87.9) |  |
| SSS NC | 73.3  (60.3 – 83.9) | P = 0.791 | 63.7  (55.3 – 71.5) | P < 0.001* | 66.5  (59.6 – 72.9) | P < 0.001* |
| SSS AC | 78.0  (65.3 – 87.7) | P = 0.267 | 67.8  (59.6 – 75.3) | P < 0.001* | 70.7  (64.0 – 76.9) | P = 0.001* |
| SDS NC | 81.4  (69.1 – 90.3) | P = 0.118 | 44.1  (35.9 – 52.6) | P < 0.001* | 54.9  (47.8 – 61.9) | P < 0.001* |
| SDS AC | 93.1  (82.3 – 98.1) | P = 0.003* | 44.1  (35.9 – 52.6) | P < 0.001* | 58.1  (51.0 – 65.0) | P < 0.001* |
| S-TPD NC | 73.3  (60.3 – 83.9) | P = 0.804 | 70.5  (62.5 – 77.8) | P < 0.001* | 71.4  (64.7 – 77.4) | P = 0.001* |
| S-TPD AC | 71.2  (57.9 – 82.2) | P = 1.000 | 69.9  (61.7 – 77.2) | P < 0.001* | 70.2  (63.5 – 76.4) | P = 0.001* |
| I-TPD NC | 71.2  (57.9 – 82.2) | P = 1.000 | 64.6  (56.2 – 72.4) | P < 0.001* | 66.5  (59.6 -73.0) | P < 0.001* |
| I-TPD AC | 76.3  (63.4 – 86.4) | P = 0.454 | 63.0  (54.6 – 70.9) | P < 0.001* | 66.8  (59.9 – 73.2) | P < 0.001* |

Values are n (95% confidence interval).

AC = attenuation correction; I-TPD = ischemic total perfusion deficit; NC = noncorrected; SDS = summed difference score; SSS = summed stress score; S-TPD = stress total perfusion deficit. *Indicating a significant difference with expert visual analysis (P < 0.05).

**Supplemental table 2:** Diagnostic performance of expert visual analysis and automated analysis using optimized software, for the detection of coronary artery disease as defined by stenosis ≥ 70% in the validation cohort (n = 103).

|  | Sensitivity | Difference with expert | Specificity | Difference with expert | Diagnostic accuracy | Difference with expert |
| --- | --- | --- | --- | --- | --- | --- |
| Expert | 69.0  (49.2 – 84.7) |  | 87.8  (78.2 – 94.3) |  | 82.5  (73.8 – 89.3) |  |
| SSS NC | 65.5  (45.7 – 82.1) | P = 1.000 | 79.7  (68.8 – 88.2) | P = 0.180 | 75.7  (66.3 – 83.6) | P = 0.189 |
| SSS AC | 78.6  (59.1 – 91.7) | P = 0.508 | 68.9  (57.1 – 79.2) | P = 0.004* | 71.6  (61.8 – 80.1) | P = 0.071 |
| SDS NC | 67.9  (47.7 – 84.1) | P = 1.000 | 79.5  (68.4 – 88.0) | P = 0.118 | 76.2  (66.7 – 84.1) | P = 0.210 |
| SDS AC | 77.8  (57.7 – 91.4) | P = 0.508 | 69.9  (58.0 – 80.1) | P = 0.004* | 72.0  (62.1 – 80.5) | P = 0.071 |
| S-TPD NC | 58.6  (38.9 – 76.5) | P = 0.453 | 87.8  (78.2 – 94.3) | P = 1.000 | 79.6  (70.5 – 86.9) | P = 0.607 |
| S-TPD AC | 82.1  (63.1 – 93.9) | P = 0.289 | 52.7  (40.8 – 64.4) | P < 0.001* | 60.8  (50.6 – 70.3) | P < 0.001* |
| I-TPD NC | 53.6  (33.9 – 72.5) | P = 0.289 | 74.3  (62.8 – 83.8) | P = 0.031* | 68.6  (58.7 – 77.5) | P = 0.009* |
| I-TPD AC | 85.7  (67.3 – 96.0) | P = 0.180 | 54.1  (42.1 – 65.7) | P < 0.001* | 62.7  (52.6 – 72.1) | P = 0.002* |

Values are n (95% confidence interval).

AC = attenuation correction; I-TPD = ischemic total perfusion deficit; NC = noncorrected; SDS = summed difference score; SSS = summed stress score; S-TPD = stress total perfusion deficit. *Indicating a significant difference with expert visual analysis (P < 0.05).

**Supplemental figure 1: Standard software ROC curves for ≥70% stenosis.**

**
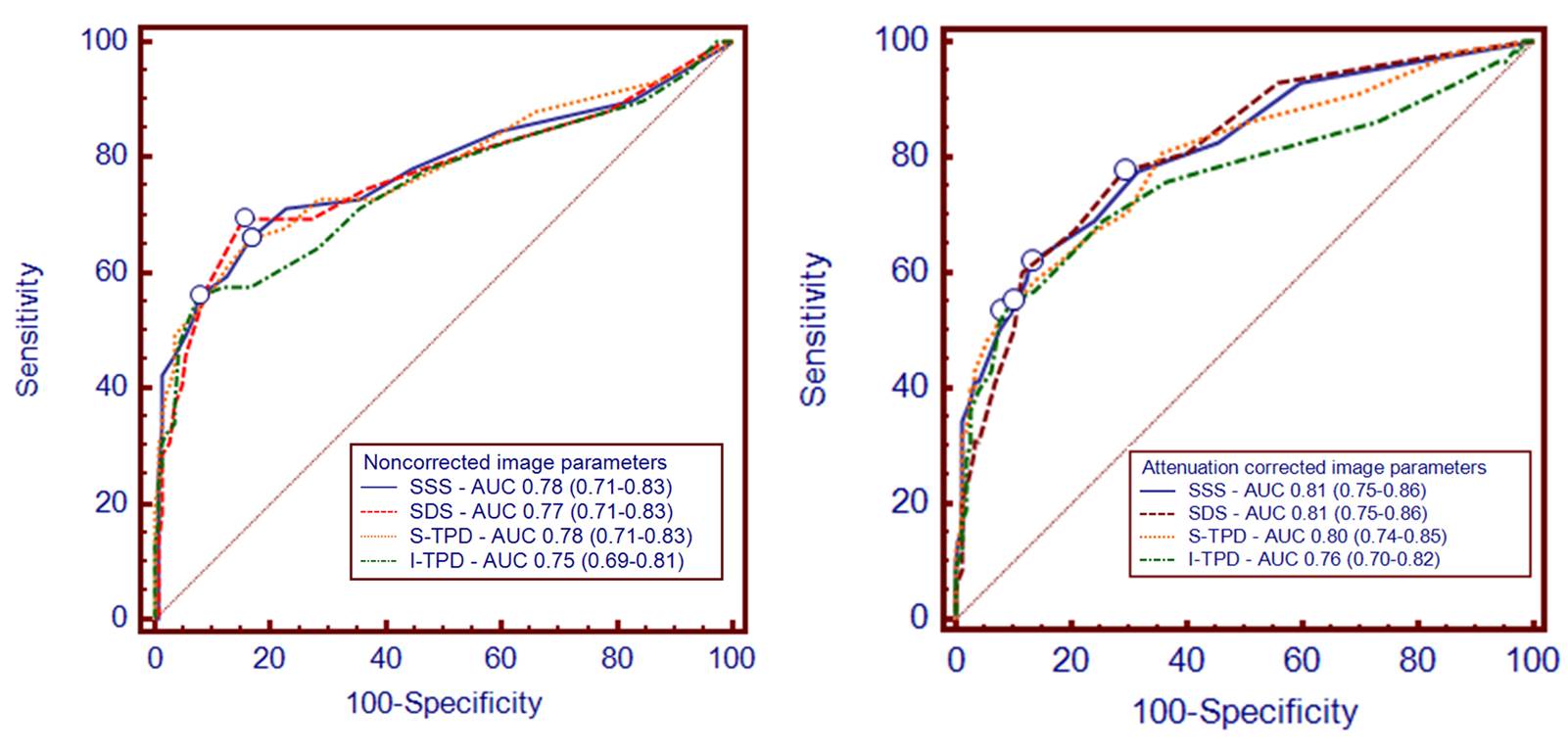
**

**Supplemental figure 2: Optimized software ROC curves for ≥70% stenosis in validation cohort.**


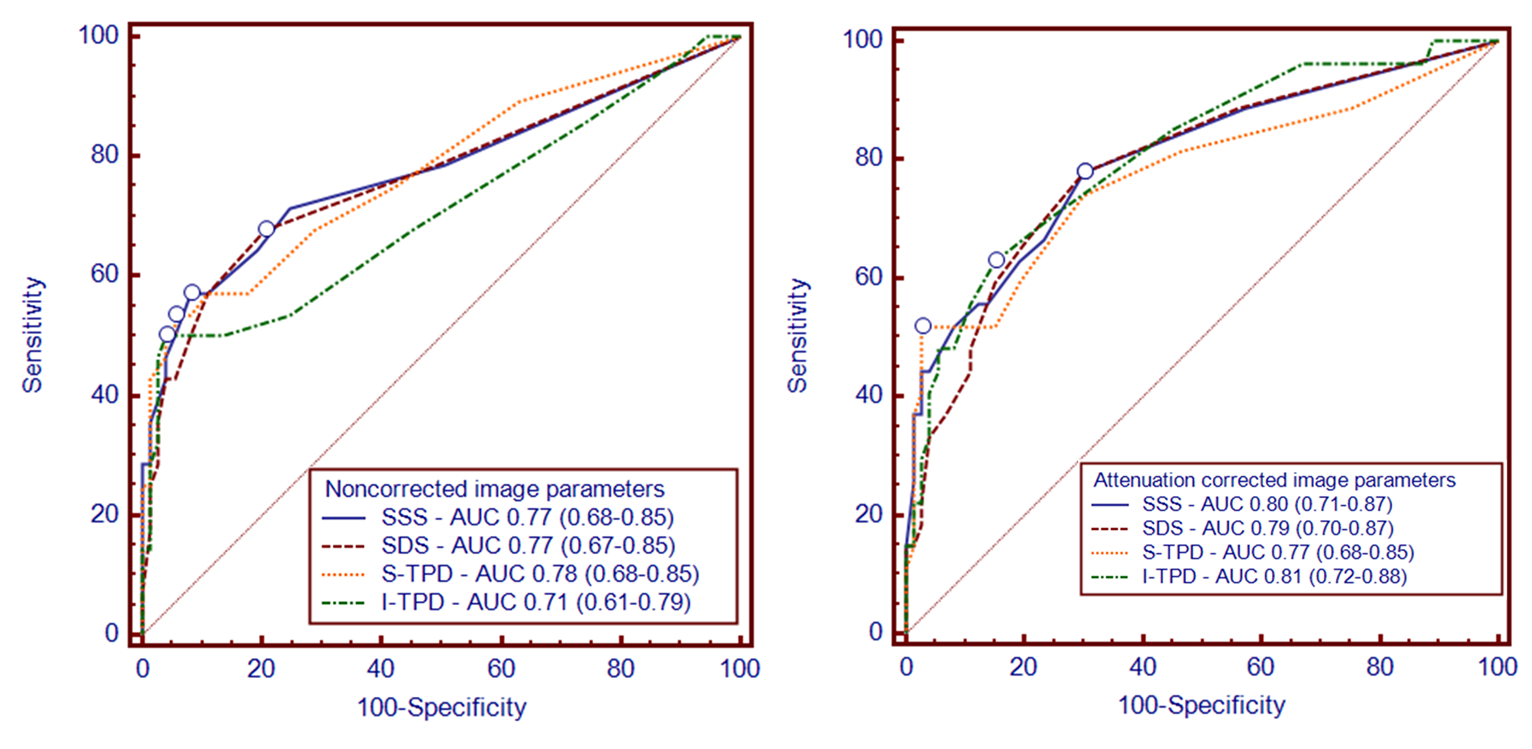


**Supplemental figure 2: Diagnostic performance in the validation cohort before and after optimization.**


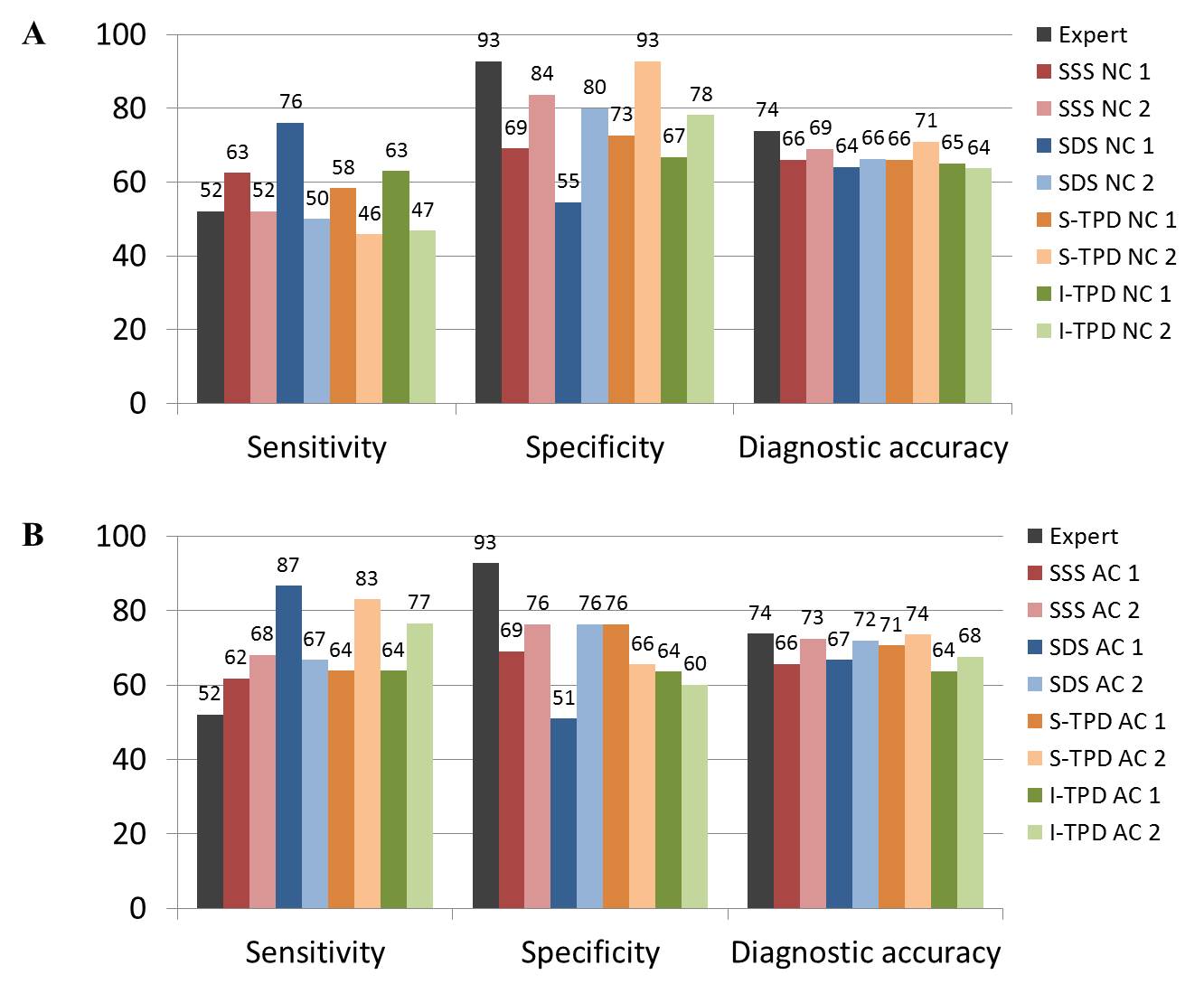

Supplement: Supplementary file 1 — (DOCX 582 kb) [file 259_2018_3951_MOESM1_ESM.docx]
